# Living Cell as a Self-Synchronized Chemical Reactor

**DOI:** 10.1021/acs.jpclett.4c00190

**Published:** 2024-03-25

**Authors:** Robert Hołyst, Grzegorz Bubak, Tomasz Kalwarczyk, Karina Kwapiszewska, Jarosław Michalski, Marta Pilz

**Affiliations:** Institute of Physical Chemistry, Polish Academy of Sciences, Kasprzaka 44/52, 01-224 Warsaw, Poland

## Abstract

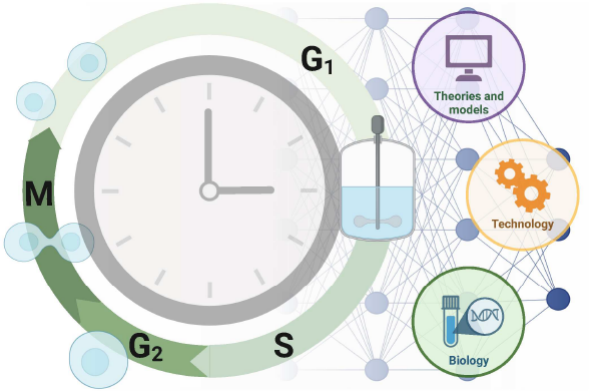

Thermal fluctuations power all processes inside living cells. Therefore, these processes are inherently random. However, myriad multistep chemical reactions act in concerto inside a cell, finally leading to this chemical reactor’s self-replication. We speculate that an underlying mechanism in nature must exist that allows all of these reactions to synchronize at multiple time and length scales, overcoming in this way the random nature of any single process in a cell. This Perspective discusses what type of research is needed to understand this undiscovered synchronization law.

## Introduction

Chemical engineers design reactors to host few reactions, usually producing vast amounts of the desired product measured in metric tons. On the contrary, living cells host myriad reactions with small amounts of substrates, leading to small quantities of products. The reactor size itself also varies significantly between the industrial and living world. For instance, the largest Sinopec reactor for a hydrogenation reaction has a volume of 2000 m^3^, while the most miniature reactor inside a cubic micrometer made by propionyl-CoA synthase has a volume of only 33 nm^3^.^1^ Modern chemical reactors allow for precise control over the external thermodynamic parameters but not over the reactions occurring inside the reactor. Living cells cannot control the external temperature, pressure, pH, and other parameters. Instead, they maintain dynamic control over parameters inside the reactor, adapting to the changes in external parameters to fulfill their goal, self-replication. All reactions inside living cells are synchronized, allowing substances to be produced without any unnecessary byproducts. This tight control enables the synthesis of complex biopolymers such as proteins and mRNA, while the best-known controlled polymer synthesis in chemical reactors (i.e., atom transfer radical polymerization) is far from achieving such precision. Living cells produce macromolecules from the substrates and use these substrates to synthesize parts of reactors and the necessary tools for fine-tuning, compartmentalization, and advanced biosynthesis steps.

In a chemical reactor, all chemical reactions leading to valuable products are thermodynamically favorable; i.e., their Gibbs free energy decreases. In living cells, many reactions run uphill; many downhill and uphill reactions couple together to afford products with thermodynamic potentials higher than those of substrates. An example of a downhill reaction is the hydrolysis of ATP, which releases energy during the transformation of ATP into ADP. The reverse is the uphill reaction, i.e., the production of ATP from ADP by the enzyme ATPase with proton current through the membrane, powering this reaction.

ATPase and kinesin exemplify the high efficiency of the living cell reactor. Both are molecular motors performing work at the expense of energy. Their efficiency is close to the theoretical limit set by the Carnot cycle. Surprisingly, their design principle is far from what we know from engineering. The good reason is that they operate in a bath of thermal fluctuations, where water molecules at a speed close to the speed of sound randomly hit biomolecules that perform the work. The artificial molecular motors (Nobel Prize 2016) with macroscopic engineering design principles attain efficiency 1 billion times smaller than that of kinesin or ATPase. One of the reasons for this considerable difference in efficiency is the design of artificial molecular motors to go against thermal noise at the expense of energy. At the same time, biological engines use thermal noise to perform work. The latter design principle is that of molecular ratchets. The motion of motors brings us to the problem of transport in cells.

In chemical reactors, flows set by external devices are the primary means of transport that mix reactants and bring them together. In contrast, in living cells, the primary means of transport is diffusion, a motion of molecules powered by thermal fluctuations. This type of motion is random. Thus, all processes occurring inside the cells are random. Why are millions of random reactions co-occurring in the cells so fine-tuned and well-orchestrated? What is the primary mechanism assembling all of these randomly occurring reactions in a single small biochemical living reactor, making them a full-size symphony orchestra that eventually leads to the replication of the reactor itself?

Available scarce quantitative data on reactions in living cells (e.g., the motion of kinesin, ATPase rotations, or propionyl-CoA synthase action) point to synchronization as the primary mechanism, allowing an assembly of myriad random processes in cells to become a chain of well-orchestrated reactions. The law of synchronization probably underlines all processes out of equilibrium, including living cells. To understand how synchronization emerges as the mechanism gluing all reactions together in cells, we have to gather data on the diffusion of reactants, reaction rates, and equilibrium constants of various reactions and create new nanoscale sensors for pH, ionic strength, temperature, osmotic pressure, and other parameters. The law of synchronization has yet to be discovered. If we do find it, so what?

In this Perspective, we try to look at a cell as a self-synchronized biochemical reactor to stimulate searching for the law of synchronization. We aim to demonstrate synchronization as a key factor in keeping biological cells alive and its lack as a general source of diseases or dysfunction. First, to better understand the objective, we summarize the necessary physicochemical properties of living cells and compare them to those of classical reactors. Next, we discuss time scales within the single cell and show synchronization examples based on available quantitative data, including some of the most important cellular processes: energy storage thanks to ATP synthases or transport of cargoes by kinesin motor protein. In a complementary manner, the transport through the cell membranes is analyzed as its disturbance can desynchronize biochemical reactions running inside our hypothetical reactor. Finally, we consider the limitations of the current biophysical methods and define challenges for developing new techniques for gaining insights into the reactions inside cells. We debate challenges arising from fluorescent methods, which allow the observation of single biomolecules in the interior of a single living cell, and some perspectives on label-free observation based on Raman scattering.

The rewards for the comprehension of a single-cell action based on synchronization will be substantial. Most probably, synchronization would reveal many mysteries about cells, the basic units of life, and how cells assemble into higher-order structures. The synchronization law would help construct synthetic living cells from organic compounds, generating clean energy, or understanding diseases. Furthermore, the one-pot synthesis of thousands of valuable products could be within reach if only we had a law telling us which reactions could be synchronized in a single pot.

## Reactor Characteristics

**Figure 1:**
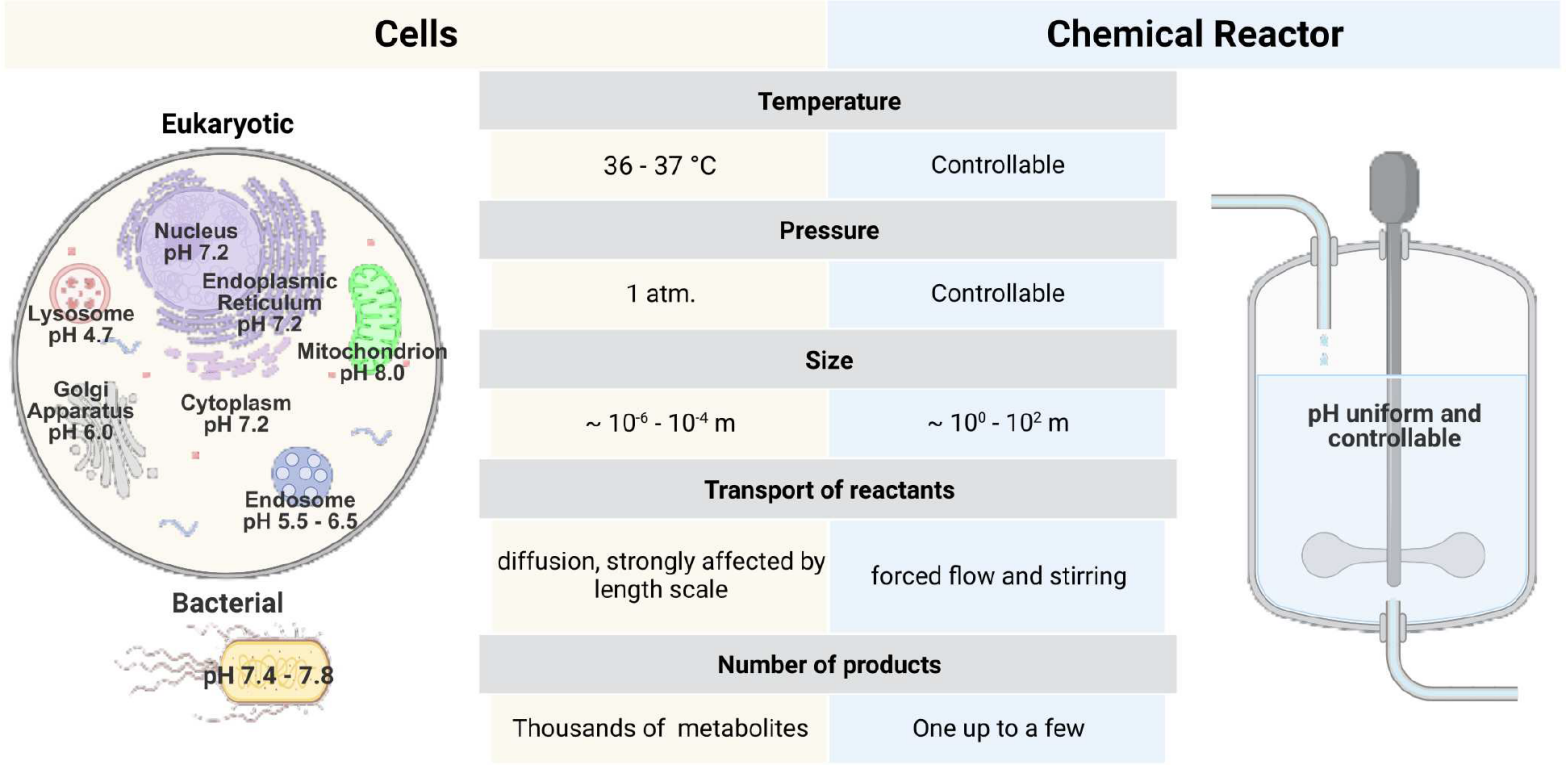
Eukaryotic and bacterial cells as self-replicating reactors vs chemical reactors. Comparison of selected parameters: temperature, pressure, size, transport of reactants, and number of products.^2−5^

Figure 1 shows the main contrast between the industrial and self-replicating organic reactors (i.e., living cells). The primary distinction between these two worlds lies in their goals. Chemical engineers usually design reactors that produce only a few substances, and processes are optimized primarily for economic reasons to achieve the highest reaction yield with the lowest possible level of resource use. In contrast, ∼1 billion biochemical reactions occur per second in living eukaryotic cells. The main goal of natural reactors is to perform these vast numbers of chemical reactions in a synchronized way, leading to replication. Moreover, the building blocks of cells are limited to organic compounds (such as peptides, proteins, nucleic acids, and carbohydrates) and limited inorganic compounds, such as ions or salts. Considering the cell as a biochemical reactor, it is challenging to combine all of these requirements at average length scales of 1 μm for bacteria (*Escherichia coli*) to 150 μm for human oocytes,^2^ with a typical volume ranging from 1 fl to 4 nl, respectively.^2^ Other parameters are also limited by the inherent properties of the biologically active molecules. For instance, the concentration of hydrogen ions plays a significant role in all crucial processes occurring in living cells, as they are based on protein interactions. It affects, for example, protein folding and unfolding, enzymatic activity, and ATP synthesis.^2^ The nonphysiological pH will impair many biologically significant processes. For example, enzymes will take altered conformations, causing a loss of their catalytic activity, dysregulation of the key and lock mechanism. Also, highly acidic or alkaline conditions will cause hydrolysis of ester and peptide bonds such as the hydrolysis of ATP to ADP or protein degradation (proteolysis). Moreover, pH values are compartmentalized within cells and differ between cellular structures and organelles to create conditions for specific reactions. In the case of eukaryotic cells, most cell reactions occur at pH 7.2, as these are reported values for the cytosol and nucleus^3^ (due to permeability). For instance, proteins are folded at a pH of 7.2 in the endoplasmic reticulum.^3^ Conversely, a lower pH of 6.0 is present in cis-Golgi cisterns and trans-Golgi networks.^3^ The lowest pH (i.e., ∼4.7) is in lysosomes,^3^ the modules responsible for “cleaning” unwanted byproducts or digesting invaders such as bacteria or viruses to minimize disturbance of the well-synchronized orchestra of reactions. The compartmentalization of pH within the cell is connected to certain tasks, resulting in optimal pH ranges for specific enzymes and reactions. That is why mitochondria generally have a pH of 7.8–8.0,^3^ but their intermembrane space has a pH of 7.0–7.4^3^ due to the higher proton concentration.^3^ Similar variety is found for endosomes, depending on their stage and function; the pH is 6.3 in early endosomes, 6.5 in recycling endosomes, and 5.5 in late endosomes.^3^ It is noteworthy that the intracellular buffering capacity results from phosphate and bicarbonate ions and other weak acids and bases. In comparison, bacteria such as *E. coli* have an intracellular pH in the range of 7.4–7.8 under the optimal conditions^3^ and do not need varying pH ranges due to their simplified architecture and a small number of chemical reactions in comparison to those of eukaryotic cells.

One of the crucial synchronization factors influencing the transport of metabolites and the rates of biochemical processes occurring in cells is diffusion, as diffusion-limited reactions are the fastest possible reactions in cells. Because most of the cell metabolism necessary to replicate our hypothetical reactor is organized by enzymes, the rate-limiting step in the entire synchronization of processes is the diffusion of the substrate into the active site or product from it. Equation 1 shows a rate constant of a diffusion-limited reaction.^6^
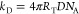
1where *k*_D_ is the rate constant of the diffusion-limited reaction, *R*_T_ denotes the effective target’s radius, *D* is the effective diffusion coefficient of the reagent, and *N*_A_ is Avogadro’s number. Equation 1 presents the simplest case with the target size being much larger than the size of the reagent.

**Figure 2:**
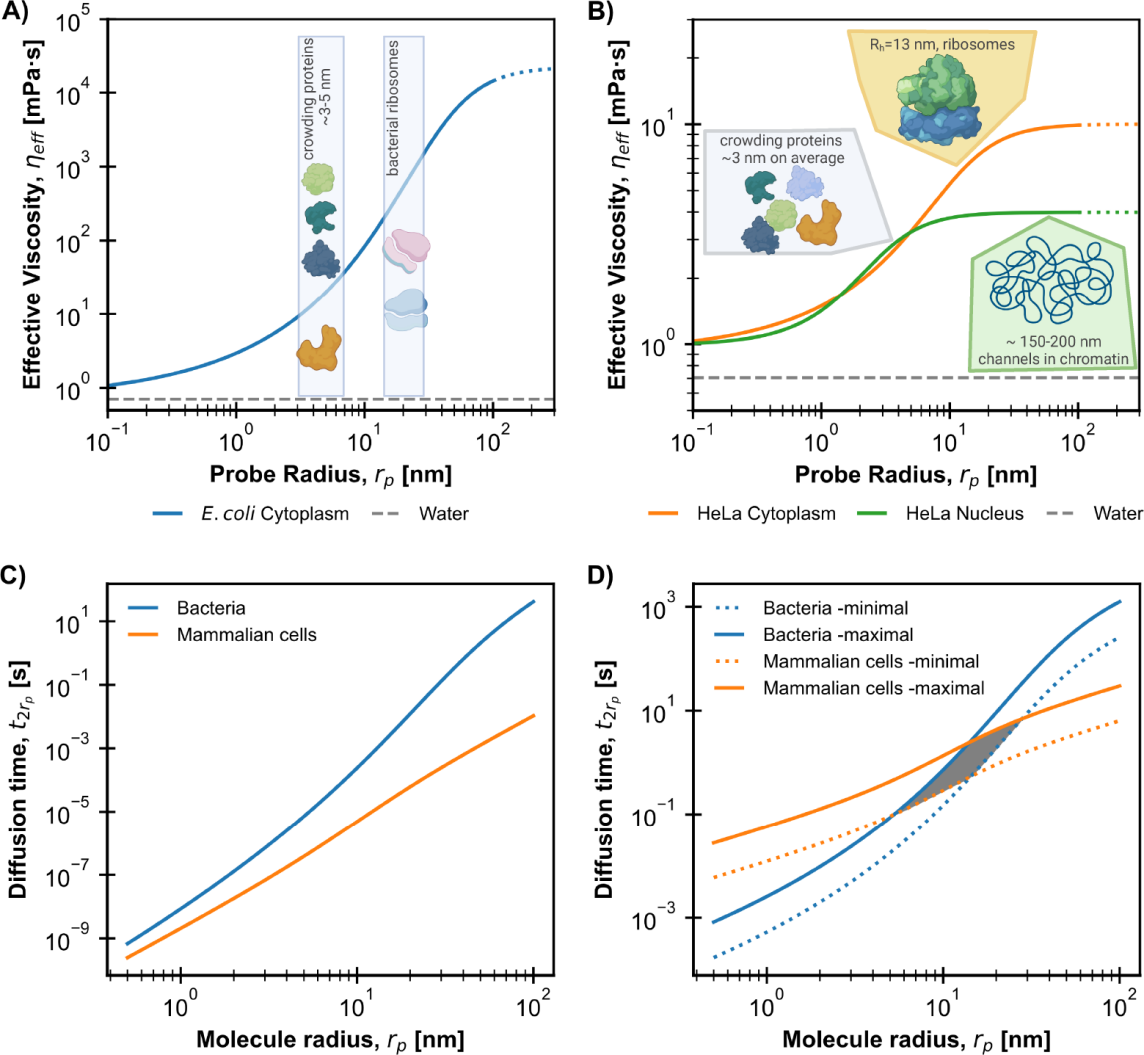
Adaptation of nanoscale viscosity in cells^5,7^ and bacteria^8^ to various sizes of diffusing biomolecules. All values were calculated for 36 °C. (A and B) Comparison of diffusion time as a function of molecular size in two types of cells. Illustrations correspond to the main molecular crowders and components affecting the viscosity on specific length scales. The plots were calculated using parameters of the length scale-dependent viscosity model (eq 2) obtained for several kinds of human cancer cells (see Figure 2 of ref (9)) and bacterial cells (see ref (8)). (C) Time needed to diffuse over the distance of the molecular diameter. (D) Diffusion time over the cell diameter’s distance. Blue curves correspond to the smallest and largest bacterial diameters (0.5 and 1.1 μm, respectively), while orange lines correspond to mammalian cells’ smallest and largest diameter (4.9 and 10.6 μm, respectively). The gray-shaded area represents the range of molecular sizes where diffusion times are similar, irrespective of cell type.

The living cell is a rather crowded environment; biomolecules can occupy up to 40 wt % of the cell interior. As a consequence, the viscosity directly affecting diffusion differs significantly from water or buffer viscosity. Moreover, our recent experimental–theoretical works have laid the groundwork showing that this viscosity is dependent on size and can be described by effective viscosity^4,5^ as shown by eq 2.
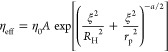
2where η_0_ corresponds to the viscosity of the solvent (water), *A* and *a* are constants on the order of unity, and ξ and *R*_H_ are characteristic length scales of a complex system. The effective viscosity experienced by molecules inside bacteria can be orders of magnitude higher than in the cytoplasm of mammalian cells. In this way, chemical reactions are somehow controlled and synchronized inside living cells. For instance, in the cytosol and nucleus, the smallest subnanometer molecules, such as sugars and inorganic ions, are as mobile as in water solutions. Therefore, access to one of the most abundant reactants is easy and common in the cellular environment. We can compare it to the continuous stream of substrates in flow reactors. On the contrary, objects ∼100 nm and even micrometers (such as large organelles) feel gel-like viscosity, which hinders their mobility within the cell and can be similar to reactors with a fluidized bed or other types of reactors using solid reactants and catalysts. Major reactants, proteins between 2 and 5 nm in size, move in the double and triple viscosity of water, which allows the necessary time for these molecules to react, orient, and combine into larger multiunit structures (see Figure 2). In addition, the different cellular compartments vary in composition and reactions that run inside, as does their effective viscosity. A more detailed discussion of viscosity, diffusion, and time scales is presented below.

## Time Scales and Synchronized Reactions in Cells

The synchronization of chemical reactions in living cells is closely tied to the time scales of various processes within the cell, such as diffusive transport, genetic material duplication, cell cycle, transcription, and translation. Hence, detailed knowledge of those and other relevant time scales is of critical interest for understanding, predicting, and controlling the living cell as the chemical reactor. Here, we briefly describe only a few of the time scales (see refs (10) and (11) for more details).

*Replication Times, Intracellular Rates, and Time Scales*. The time scales of intracellular processes vary between cells. Here, we will discuss the differences between human cells and bacteria, keeping in mind that human cells are biomedically important while bacteria are valuable in biotechnology. The self-replication of the reactor time scale is closely related to the cell cycle, which takes days in mammalian and human cells. During this process, cells replicate their genome at a rate of ∼40 bases per second. The copying of the proteome is performed at rates limited by two steps: the transcription of the genetic information to mRNA (approximately 10–100 nucleotides per second) and its translation into proteins (approximately 10 amino acids per second). The time scale responsible for controlling changes in the concentrations of substrates and products is associated with the metabolites’ half-life turnover, which takes ∼1 min.

In contrast, the bacterial cycle takes minutes, during which cells replicate their genetic information, including the genome and proteome. The genome replication rate reaches 1000 nucleotides in seconds. However, despite the genome length (4.6 million nucleotides for *E. coli*), bacterial cell division typically takes only ∼20 min, ∼4 times faster than one can calculate from the rates. The discrepancy results from the simultaneous performance of multiple replication rounds.^12^ The transcription/translation rates in bacteria are at the same level as those in mammalian cells. The half-life of metabolites undergoing turnover is ∼1 s.

All of the time scales discussed above are longer for mammalian cells than for bacteria. However, the time scale associated with the diffusive motion of molecules inside the cell cytoplasm is at odds with the others. To visualize it, we will perform some calculations. Referring to the effective viscosity concept described by eq 2, we calculated the diffusion coefficient as a function of the size of the particle undergoing translational diffusion. We used the Stokes–Sutherland–Einstein formula and the viscosity defined by eq 2 to calculate the diffusion coefficient (at 310 K): *D* = *k*_B_*T*/ζ, where ζ = 6*πη*_eff_(*r*_p_)*r*_p_. Next, we calculated the time required for a molecule to travel a distance equal to its diameter (*L* = 2*r*_p_): τ_2*r*_p__ = *L*^2^/(6*D*). We compared diffusion times for bacteria and mammalian cells in Figure 2C. Depending on the molecule’s size, the diffusion times in bacteria can be >2 orders of magnitude longer than in human cells.

On the contrary, τ_*r*_cell__, the time scale of diffusion at the distance of the cell radius (mammalian cells are ∼10 times larger than bacteria), is longer for mammalian cells than for bacteria for molecular sizes up to ∼10 nm (see Figure 2D). In the range of molecular sizes from 10 to 40 nm, comparable to the size of ribosomes, there is a time scale window in which diffusion times across the cell are comparable and between 1 and 10 s. Such a long diffusion time makes the ribosomes nearly immobile from the perspective of metabolite molecules and small proteins.

The comparison of the diffusion times in bacteria and human cells gives counterintuitive results when compared to other time scales. As we show further, however, the processes based on diffusion are the most important for synchronizing some physicochemical processes in cells.

*Examples of Synchronized Biologically Relevant Reactions*. A unicellular or multicellular organism operates correctly only when its biophysicochemical machinery remains synchronized. Desynchronization of the given process by internal or external stimuli can lead to various dysfunctions and disorders. Here, we provide examples of two processes in which the chemical reactions, which are the foundation of these processes, are well synchronized. We describe the mechanisms, provide crucial intracellular time scales, and briefly describe the results of the desynchronization of the processes.

**Figure 3:**
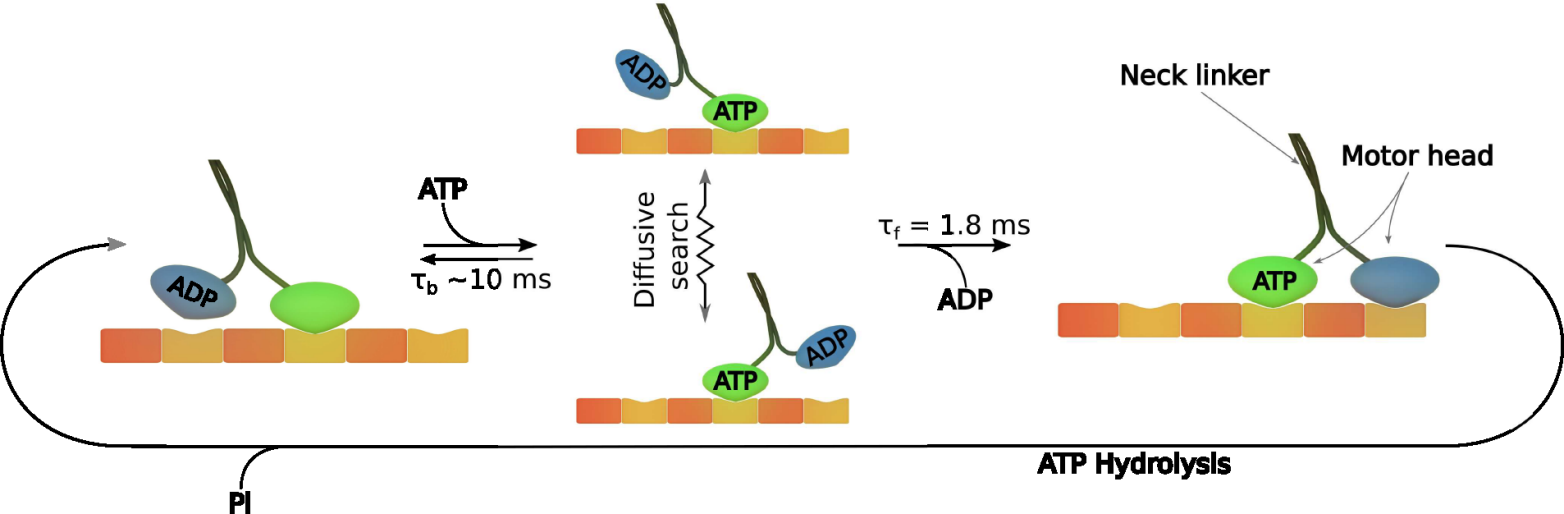
Scheme of the mechanism of kinesin-1 motion. Initially, the kinesin motor is in the ATP-awaiting step. After the attachment of ATP, the structural changes promote free diffusion of the ADP-bound head. At this time, the head is searching for a new binding site. Under the *in vitro* conditions (in aqueous solutions), the step forward takes ∼1.8 ms. The step backward, connected to the release of ATP, is ∼5-fold longer. For example, the diffusive motion can be elongated by the increased viscosity locally experienced by the kinesin head. In such a case, the diffusion time becomes comparable to τ_b_, and the kinesin stalls. Finally, the release of ADP is connected to the attachment of the diffusing head to the microtubule, followed by release of the phosphate ion.

Kinesin-1. The directed motion of the kinesin-1 molecular motor is an excellent example in which mostly unidirectional motion results from the randomness of diffusion and time scale balance of chemical reactions occurring at every step of the mechanism. Kinesin-1 is a molecular motor responsible for active transport within eukaryotic cells. It travels along microtubules, transporting cargo ranging in size from nanometers to micrometers. *In vitro*, outside the cell, the kinesin motor can travel along the microtubule at an average velocity of ∼800 nm/s. Figure 3 represents the process scheme. The motor spends most of its time in the ATP-awaiting state. When the ATP molecule attaches to the motor domain bound to the microtubule, the neck linker’s orientational freedom changes, causing the unbound motor domain to diffuse. The linker needs to be extended by ∼3 nm before the motor domain finds its new tethering position, which takes around 1.8 ms (τ_f_^aq^). The same motor head can return to the old binding site, which takes ∼10 ms (τ_b_). The whole stepping mechanism depends on the relation between these two time scales.

We recently showed^13^ that the mechanism can be desynchronized when the kinesin travels in a solution of low-molecular weight crowders. Under such conditions, the viscosity effectively felt by the motor domain, η_eff_, was only 5-fold greater than the water viscosity, η_0_. The step-forward time, however, was increased to ∼8.8 ms.^a^ The motor stalled because the time required for a diffusive finding of the new binding site became comparable to the time of unbinding of ATP. The neck linker takes the initial conformation when the ATP dissociates from the head. The motor is waiting for ATP. Therefore, when the diffusion of the motor head is hindered, two processes controlled by ATP attachment and disatachement start to compete, resulting in a nearly 50/50 chance of moving or stalling in every step.

At this point, it is instructive to analyze how the kinesin motor performs in a much more complex environment, the cytoplasm of a living cell. Again, we used the model of length scale-dependent viscosity but with the parameters obtained for living U2OS cells.^4,9^ We chose the U2OS cells because of the availability of literature data for kinesin motion in this particular cell line.^14^

The hydrodynamic radius of the kinesin head that we placed is equal to 2.5 nm. According to the length scale-dependent viscosity model, the viscosity that is effectively felt by the kinesin head in the cytoplasm (η_eff_) is 2.64 ± 0.65 mPa s, which is ∼2.9 ± 0.7 mPa s higher than η_0_. Keeping in mind that τ_f_^cyto^/τ_f_^aq^ ∝ *D*^aq^/*D*^cyto^ ∝ η_eff_/η_0_, we found the expected τ_f_^cyto^ in the cytoplasm of U2OS cells equals 4.6 ± 1.4 ms.

Another valuable insight is the recent data obtained using the modern super-resolution MINFLUX technique. The technique allows the localization of fluorophores with a few-nanometer precision and a time resolution down to one-tenth of a millisecond. In the literature, one can find the data for tacking the kinesin-1 *in vitro*(15) and *in vivo*.^14^ The high temporal resolution allowed researchers to observe stepwise time traces of kinesin-1 motion. The dwell time needed to make the complete step forward (τ_16 nm_^aq^) (both heads, distance *L* of 16 nm), observed under *in vitro* conditions, was ∼8.75 ± 1 ms. Even more interesting are the dwell time data obtained *in vivo* from three-dimensional tracking of kinesin-1 in U2OS living cells utilizing the MINFLUX. Here, the dwell time (τ_16 nm_^cyto^) equals 26.3 ms (cf. Figure S2 of ref (14)). Keeping in mind that the step forward is realized by the diffusive motion (τ_f_ = *L*^2^/6*D*), we recalculated the literature values to obtain the time required for diffusive traveling at a distance of 8 nm (single head). We obtained a τ_8 nm_^aq^ of 2.19 ms and a τ_8 nm_^cyto^ of 6.6 ms for the *in vitro* and *in vivo* data, respectively. Conversely, the experimentally measured dwell time for the single step is 3.7 ms, which is closer to the value predicted by the length scale-dependent viscosity. The discrepancies between the experimentally measured and calculated values of the *in vivo* dwell time are likely due to the high variance of the experimental data.

In living organisms, desynchronization of the kinesin-1 motor leads to severe disorders. For example, point mutations in the KIF5A gene reduce the affinity for microtubules and/or motion velocity or even stall the motor as a whole. Those disruptions of the molecular mechanism cause the neurodegenerative disorder called hereditary spastic paraplegia, which manifests as slow and progressive lower limb paralysis.^16^

**Figure 4:**
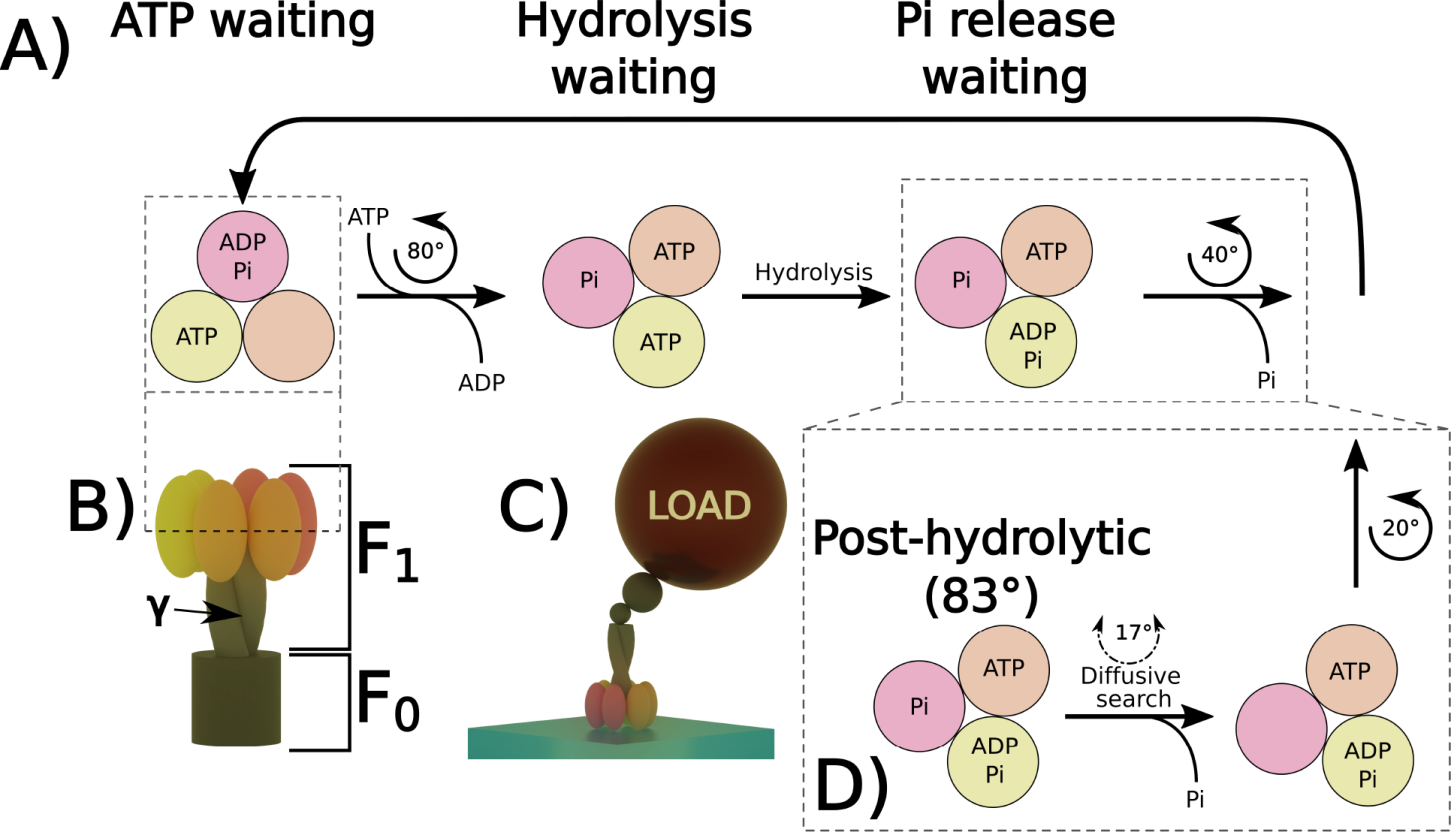
(A) Scheme of the rotational motion of the ATPase F_1_ motor as proposed on the basis of cryo-electron microscopy measurements.^17^ After ATP is attached, the protein complex rotates by ∼80° and releases an ADP molecule. Next is a hydrolysis waiting step, followed by the phosphate release step and further rotation by ∼40°. (B) Scheme of the molecular structure of ATP-synthase composed of two motor domains, F_0_ and F_1_, and the γ shaft connecting both motors. (C) F_1_ motor attached to the solid substrate. The γ shaft is loaded with nanoparticles or microtubules (see refs (18) and (19)). (D) Proposed step of the F_1_ rotation mechanism, including the search for the proper conformation through rotational diffusion. The model was inspired by the models described in refs (17) and (20).

F_1_ ATP-Synthase Motor. Another example of a synchronized molecular motor is the F_1_ domain of the ATP synthase complex. The whole ATP-synthase complex combines two competing rotary motors (Figure 4B). The first one, F_0_, is responsible for consuming the ADP needed to synthesize ATP in response to the transmembrane electrochemical gradient. The second motor, F_1_, operates against F_0_ and causes ATP hydrolysis to generate the motor’s rotational motion, pumping protons against the chemical gradient. The mechanism of F_1_ action remains unclear and has been studied through static and dynamic approaches. In the static approach, the ensemble of proteins is frozen, and the conformations taken by proteins are analyzed using electron microscopy imaging. From this approach, we learn that the full rotation of the F_1_ motor is realized in three steps, 120° each, and requires three ATP molecules. Each of the 120° steps can be further divided into several substeps^17^ (see also Figure 4A).

In the dynamic approach, the F_1_ domain is typically isolated and fixed on a solid substrate. Moreover, the γ subunit that connects the F_0_ and F_1_ domains carries a high-hydrodynamic drag object such as nanoparticles or microtubules. This technique allows for the observation of motor rotations by monitoring a much larger object, as shown in Figure 4C. The intriguing thing is that the F_1_ motor can still function even when it is subjected to very high loads, such as when it is loaded with actin filaments.^18^ In such cases, the motor experiences hydrodynamic drag that is 2 × 10^5^ to 10^7^ times higher than that experienced by an unloaded motor.^b^ However, even a small countertorque (0.15*k*_B_*T*) exerted on the γ subunit by the F_0_ motor can stop the F_1_ motor from functioning.^20^

Kulish et al.^20^ proposed a Brownian ratchet-like^22^,^c^ model to describe F_1_ motor rotation, analogous to the mechanism proposed for kinase-1. The difference is that the rotational motion plays the first fiddle in F_1_ motor rotation, while the translational diffusion of the head limits the step forward for kinase. The rotor performs diffusive motion (on an angle of 120°) in the elastic potential generated by the γ shaft, and the time of diffusional searching for the new conformation is the main limiting factor of the step forward. The authors additionally assumed that the motion occurs after the ATP hydrolysis and neglected the unbinding of ADP and the phosphate, even though it is often postulated as the limiting step in the F_1_ motor rotation, with a time scale of milliseconds.^24^ Nakano et al.^17^ noticed that the phosphate release occurs within a 17° change in conformation from the posthydrolytic state (∼83°) to ∼100°. This step is followed by further rotation to 20°, during which the structure of the γ shaft relaxes. Combining Nakano’s observation with the concept of Brownian ratchet, we can postulate that the free diffusional rotation of the F_1_ rotor occurs just after the hydrolysis and is terminated by the detachment of the phosphate ion (see also Figure 4D).

Desynchronization of the ATP-synthase machinery can lead to severe (even lethal) disorders of the neuromuscular system in newborns. Desynchronization caused by gene mutation can independently influence the work of F_0_ and F_1_ motors. For example, the T89993G mutation can reduce the rate of ATP synthesis (F_0_ domain) by 50–90% while ATP hydrolysis (F_1_ domain) remains untacked. On the contrary, the T9185C mutation caused a decrease in the rate of ATP hydrolysis of 30%, keeping the ATP synthesis unaffected.^25^

Both mutations are in the domain of the F_0_F_1_ ATP-synthase complex, located in the membrane. Because F_0_F_1_ ATP-synthase is a protein that regulates the ionic balance, its desynchronization dysregulates the membrane transport, a non-negligible aspect of the cell-as-the-reactor concept, which is discussed in the next section.

## Membrane: Inlet and Outlet of the Organic Reactor by Design

All cells, whether bacteria or mammalian, are separated from their surroundings by a surface barrier. This boundary strictly regulates the inflow and outflow of molecules required for various biochemical reactions. Membrane transport, among others, ensures that reactions, like in chemical reactors, proceed with the highest efficiency toward the desired outcome: the proper function of a cell and, if needed, its replication. These would not be achieved if the cellular barrier were fully permeable to every kind of molecule because, in such a case, the cellular interior would mirror the surrounding environment. Therefore, cell membrane transport is highly selective. Understanding this selectivity of the barrier is critical for manipulating cell functions effectively by modifying the environment, which is much easier to control.

**Figure 5:**
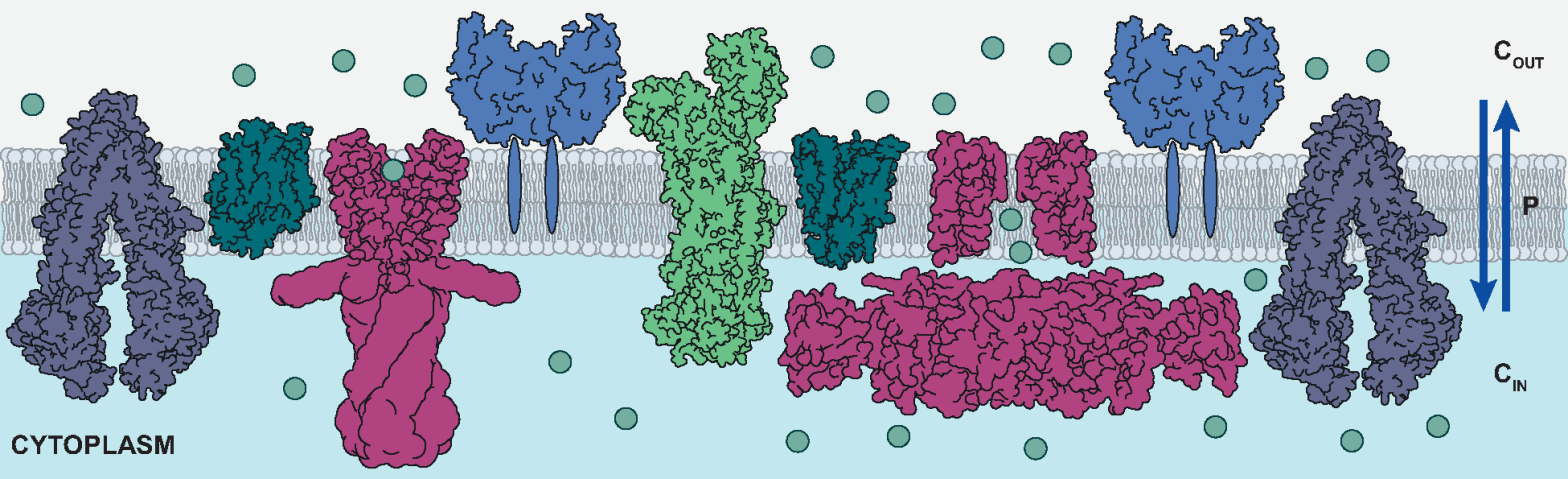
Membrane permeability coefficient, *P*, that can quantitatively describe the transport of molecules across cell membranes. This parameter characterizes how many molecules cross the membrane given the difference in concentration between the cell’s exterior, *C*_OUT_, and interior, *C*_IN_.

*Membrane Transport*. The properties of transport of molecules across the plasma membrane in eukaryotic cells can be quantified as the membrane permeability coefficient, which describes the number of molecules crossing a given membrane area per second. This measure is also known as a flux, *j*:
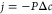
3where *P* is the permeability coefficient and *Δc* is a difference in the concentration of molecules on both sides of the membrane (see Figure 5). A higher value of coefficient *P* indicates a faster rate of molecule passage. For example, gases such as O_2_ and CO_2_ have permeability coefficients across artificial lipid membranes on the order of 10^8^ and 10^6^ nm/s, respectively.^26^ This suggests that gases permeate across membranes at a time scale similar to that of diffusion (the diffusion coefficient of O_2_ in water, at 25 °C, is ∼2 × 10^9^ nm^2^/s).^27^ On the contrary, lipoidal membranes appear to be impermeable for not much larger ions such as Na^+^ and K^+^ (with diameters of 0.095 and 0.133 nm, respectively). These cations have permeability coefficients on the order of 10^–7^ nm/s, 15 orders of magnitude lower than those of gas molecules. However, a low lipid permeability does not imply that these ions do not move across plasma membranes. Instead, it suggests a different, more controlled, selective transport mechanism.

More than one-third of the proteins in sequenced genomes are membrane proteins that form the cellular machinery for interactions with the environment. Three main categories of proteins regulate the transfer of ions and other solutes across cell membranes: pumps, carriers, and channels. They all undergo conformational changes, allowing specific molecules to pass through them. However, these membrane proteins differ in all other properties, i.e., energy input, transport rate, substrate specificity, plasma membrane distribution, and regulatory mechanisms.

As mentioned above, potassium ions cannot freely diffuse across lipid membranes but can enter cells through channels down their concentration gradient. Channels are specialized pores specific to certain ions that typically open and close in a regulated manner. While open, 10^6^–10^8^ ions per second cross the membrane through a channel. The transitions between the open and closed states occur on a millisecond time scale and can be regulated by various gating mechanisms. For instance, potassium channels can be classified into four groups on the basis of their activation: (i) calcium- and sodium-gated, (ii) voltage-gated, (iii) lipid-gated, and (iv) two-pore domain potassium channels regulated by a variety of physiological and pharmacological mediators.

Potassium ions flow across the cell membrane through channels. However, diffusion stops once a state of equilibrium is reached between the cell and its environment. Restoring the ion concentration gradient and, more critically, maintaining the distribution of K^+^ with intracellular concentrations (∼140 mM) that are ∼30 times greater than outside (∼4–5 mM) necessitates the activity of the pumps. Pumps, another group of membrane proteins, need energy to selectively transport molecules up a concentration gradient. For example, Na^+^K^+^-ATPase pumps utilize ATP hydrolysis to transport Na^+^ out of and K^+^ into animal cells. The rate at which Na^+^K^+^-ATPase pumps transfer ions across the plasma membrane is ∼100 Hz, 4 times slower than the rate at which K^+^ diffuses through channels.^28^

Pumps generate ion gradients, which serve as a source of energy for carriers. A range of carriers transport selected chemical substrates across the cell membrane. They bind substrates on one side of the membrane and release them on the other through a conformational change that repositions the binding site within a carrier. The conformational change of a carrier is a rate-limiting step with the overall transport rate ranging from 0.1 to 1000 molecules per second. Unlike pumps and channels, the binding sites of carriers exhibit intermediate specificity for substrates, and carriers can work both down and up a concentration gradient.

Carrier proteins execute the transport of the most essential nutrient, glucose. Most, if not all, mammalian cells express glucose carriers in various forms, including members of the GLUT protein family. The GLUT proteins have different distributions in tissues and cells, and depending on their localization, they exhibit various kinetic and regulatory properties. GLUT1 is expressed at the highest level by human erythrocytes, with >200 000 molecules per cell, located mainly in the plasma membrane.^29^ In contrast, GLUT4 is predominantly present in muscle and fat cells within intracellular membrane compartments but is nearly totally excluded from the plasma membrane without stimulation. Insulin, or workout in the case of muscle cells, stimulates the redistribution of GLUT4 from its intracellular sites to the plasma membrane.^30^ This example perfectly illustrates that cells control membrane transport by regulating the number of membrane proteins that serve as intermediates in the uptake process. Accordingly, a general synchronization mechanism must merge the activity of intracellular machinery with that of the cellular surroundings. It is essential to consider that membrane transport should not be viewed as an independent process for individual substances.

Moreover, during endocytosis and exocytosis, the plasma membrane is constantly redistributed. In endocytosis, cells take up various substances from the environment by engulfing them in a vesicle derived from the cell membrane. Consequently, surface molecules are also taken into account during this process. While some of these components are degraded, most are recycled back to the cell surface during exocytosis. As a result of this continuous redistribution, fibroblasts, during each hour, exchange the equivalent of 50% of their surface area.^31^

*Strategies for Exploiting Membrane Transport*. Because the cell takes up molecules on the surface, this phenomenon is used to design drugs that can bind to membrane proteins and reach their intracellular targets. In addition to drugs interacting with surface molecules, there are other examples of using membrane transport to influence cellular functions. Cells employ the synchronized action of pumps, carriers, and channels to maintain a constant volume. Regulation of cell volume is necessary because water can cross membranes via channels and lipid bilayers (permeability coefficient across artificial lipid membranes on the order of 10^4^ nm/s)^26^ as a response to changes in intracellular content or extracellular osmolarity. Changes in extracellular osmolarity induce water to flow inside or outside the cell, causing cell swelling or shrinkage. Thereby, a rapid change in medium osmolarity can be used to deliver macromolecules to the cell interior. Applying this osmotic shock in a regulated manner using a polymer-based hypertonic medium proved to be an effective way to deliver polymers, plasmids, and small nanoparticles to cells.^32^

Membrane transport analysis may provide valuable insights into the barrier that isolates the cell interior from its external environment. A thorough comprehension of this phenomenon has the potential to yield novel approaches for the efficient delivery of molecules into the cell that can reprogram a self-replicating chemical reactor (e.g., mRNA therapies) or stop it (e.g., cancer treatment).

## Challenges

Recent decades have brought unquestionable development to molecular biology. Our knowledge of biological molecular pathways and their interplay is increasing almost daily. Filling the static map of biochemical interactions with quantities, time scales, rates, and equilibrium constants is still missing and challenging. The lack of reliable data in this area is strictly related to the limits of biochemical and biophysical methods available to researchers. Analytical methods in biology can be divided into *ex vivo* and *in vivo* ones.^d^ Extraction of the analyte (*ex vivo* strategy) enables the application of highly sensitive bioanalytical techniques such as liquid chromatography (LC) or mass spectrometry (MS). However, the extraction and purification of the material can influence the sample. Moreover, this approach is suitable mainly for averaging from a population of cells; still, methods addressing single-cell extraction and analysis are being developed.^33^

**Figure 6:**
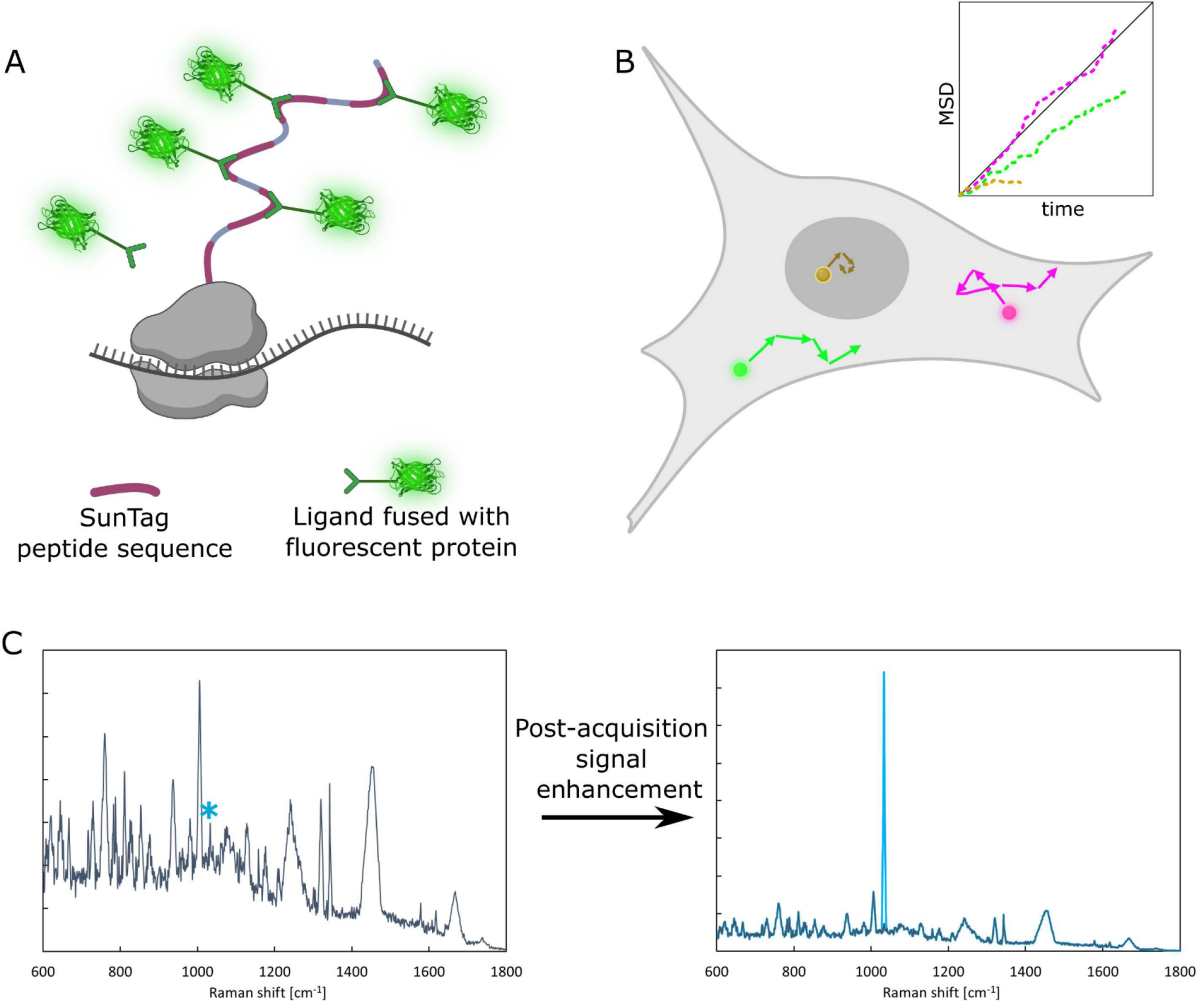
(A) Example of the in-experiment signal enhancement of a single-molecule SunTag peptide sequence fused with the protein of interest recognized by a fluorescent protein-tagged ligand. Adding multiple SunTags to the sequence enhances the signal from the single molecule. Moreover, the translation rate can be observed with this tool (adding subsequent ligands). (B) Single-particle tracking relies on microscopic observation of the movement of tagged molecules and/or nanoparticles. For each molecule, mean square displacement (MSD) data are derived, and a conclusion about diffusion coefficients or active transport rates in different cellular compartments can be derived. (C) Label-free Raman spectra of biological samples consisting of numerous peaks. The challenge is identifying peaks characteristic of the molecule of interest and postacquisition enhancement of the signal.

Alternative and more promising approaches for cellular dynamics for collecting reliable quantitative data from living cells are *in vivo* techniques. Measuring cellular processes *in vivo* requires a way to “see” the analyte. The most widespread approach is to label the molecule of interest with a fluorescent tag and detect the signal by using optical methods. The current development of detection methods, as well as tagging strategies, is leading to research works describing the observation of biochemical processes in living cells at a single-molecule resolution (SunTag for mRNA translation, FRET or FCS for protein–protein dissociation constants, etc.).^7,34^ The major challenge for these techniques is increasing the signal-to-noise ratio. The cellular interior comprises various organic molecules, exhibiting a broad autofluorescence spectrum. This background fluorescence significantly reduces the likelihood of detecting a single molecule of a fluorophore of interest. Alternatively, fluorescent labels can be multiplied to increase the intensity of the signal (Figure 6A). However, adding even a single label can affect the studied process by influencing the fragile structure of biomolecules or adding a steric restriction to molecule–molecule interactions. Moreover, the introduction of a fluorescent label can be too invasive for specific cell types. In summary, technologically fluorescence-based methods for single-molecule studies *in vivo* are still the most promising and accessible, but care must be taken with implementation.

Label-free technologies are being developed in parallel to fluorescent labeling. The challenge is to find the source of a signal that would be unique for the molecule of interest, enabling recognition of the analyte from a complex background. So far, Raman spectroscopy has been shown as a potential label-free technique that can be applied to living cells.^35^ Raman scattering is a sensitive tool for cell phenotyping, metabolic characterization, or detection of specific biomolecules. However, living cells have myriad compounds, and analyzing *in situ* Raman spectra is highly complex. What is most needed now are computing methods that enable filtering and enhance the signal of interest (Figure 6C). Also, artificial intelligence (AI) development can provide new tools for label-free bioanalysis. Strategies for analyzing AI-assisted extensive data sets seem to be able to lead to breakthroughs in this field.

The quantification of the dynamics of cellular processes *in vivo* is challenging due to the complexity of the cellular interior. Depending on the process, cells can exhibit significant variability in quantities of molecules, even among one homogeneous population.^36^ This cellular variability hinders the possibility of extrapolating data obtained at the single-cell level to whole populations of cells. A natural strategy would be to increase the sample size, which usually implies an extended time or reduced sensitivity. For example, when cellular uptake of the macromolecule is studied, flow cytometry is usually the method of choice, as it enables the screening of tens of thousands of cells in minutes. However, an increasing speed makes the technique sensitive to false positives (i.e., analyte nonspecifically bound to the membrane) or false negatives (low concentrations of the analyte or analyte removal during sample preparation). Additionally, flow cytometry is the end-point type of analysis, which means time resolution is limited to time scales of sample preparation (minutes to hours), which is not always compatible with the studied process. The alternative strategy is the application of confocal microscopy-based methods (imaging, single-particle tracking, etc.), which can address specific questions and eliminate artifacts (Figure 6B). Still, the throughput of such methods is limited (tens of cells per hour). Thus, obtaining results from a reliable number of cells is time- and labor-consuming, raising questions about such a strategy’s profitability. Therefore, the vital challenge in developing methods for the quantification of biochemical reactions is finding a balance between throughput and sensitivity. Sensitivity should be the highest priority when analyzing a few molecules in a highly complex matrix, and throughput should be increased at multiple levels: from data acquisition (hardware automation) through processing to analysis (software development).

Finally, understanding synchronization among various bioprocesses requires data on quantities of molecules and time scales of the processes. Biochemical reaction time scales range from microseconds (conformational changes in proteins)^37^ to hours (DNA replication time in human cells). This wide range of time scales implies the need for a broad spectrum of analytical methods adjusted to the time scale of the process of interest. Most biochemical reactions *in vivo* are diffusion-limited; it takes a relatively long time for the molecules to reach the appropriate proximity and orientation, and the reaction occurs immediately. Thus, it can be assumed that the most probable time scales of the reactions are within seconds and below.^4^ Naturally, subsecond temporal resolution is required to observe such processes. Adding to that spatial resolution at the length scales below micrometers (size of subcellular compartments and niches), direct observation of biochemical reactions *in vivo* seems impossible at present. Today, researchers need to choose their priority between spatial (i.e., super-resolution microscopy) and temporal resolution (i.e., ultrafast imaging). To date, no technology has been able to couple these two features. Pushing the limits of spatiotemporal resolution is an awaited breakthrough for time-resolved biochemistry *in vivo*.

## Conclusions

A few examples from our Perspective show how cells synchronize many steps in a single reaction. The prime example is kinesin motion. Without an internal linker, kinesin would move in one step (8 nm) in a few microseconds. Without ATP consumption, the average time to detach the kinesin head from the microtubule is a few minutes. The difference between these two time scales spans >7 orders of magnitude. The linker increases the diffusion time to the next spot on the microtubule from microseconds to milliseconds. The consumption of ATP reduces the time of detachment from the microtubule from minutes to milliseconds. The final synchronization tuning between detachment from the microtubule and the duration of one step makes the kinesin protein a molecular ratchet operating at the Carnot cycle efficiency. Although, by careful experimental study of various biochemical processes, we can analyze them from the point of view of synchronization, the challenge is being able to predict the global synchronization of all of them together inside a cell. The intricate synchronization of cellular processes is indispensable for cellular homeostasis and responding to environmental changes. Disruptions in the yet-unknown synchronization mechanisms contribute to cellular or even whole organism dysfunctions. An example of such dysfunctions can be lysosomal storage disorders (LSDs), a group of >70 inherited metabolic disorders caused by inefficient activity of lysosomal enzymes.^38^ In LSD, point mutation (in most cases) of a gene-encoding protein alters its structure and decreases its enzymatic activity. As a consequence, the decomposition of one metabolite is obstructed (unsynchronized), which leads to its accumulation, causing severe health problems, including organ damage and premature death.^38^ Treatment of LSDs involves enzymatic therapy, which, technically, is adjusting time scales of metabolite inflow and decay processes.^39^ We believe that exploring desynchronization as a source of disease may provide new treatment ideas and strategies.

Alternatively, desynchronization can be beneficial under certain conditions. We know that diffusion coefficients of molecules in cells remain stable during the cell cycle.^40^ However, stressful conditions can promote significant hindrance diffusion, observed for bacteria,^41^ yeast,^42^ and human cells.^43^ Thus, desynchronization seems to be a protective mechanism in the hostile environment; by stopping reactions, cells become dormant and can restore their functions when the threat is over.^41,42^

Where should we find the law of synchronization? We believe that the framework for finding new laws of synchronization for systems that are not at equilibrium is naturally a non-equilibrium thermodynamics. Non-equilibrium thermodynamics provides a theoretical framework for understanding the dynamic behavior of living cells, emphasizing the role of energy and entropy in the synchronization of cellular processes. Cells are kept in non-equilibrium states by the continuous flux of energy. They continuously grow and divide. They consume high-Gibbs free energy substrates and change them into low-Gibbs free energy products. Some recent studies point out the existence of variational principles governing the behavior of cells. According to the study of Niebel et al.,^44^ thermodynamics constrains cell metabolism and sets an upper rate limit for cellular Gibbs energy dissipation. The incredible journey toward a quantitative understanding of life at the physical chemistry level has just begun. We need a vast amount of quantitative data characterizing cell metabolism and the principles of global non-equilibrium thermodynamics^45^ for these data analyses. Thus, the law of synchronization will emerge from the application of the laws of non-equilibrium thermodynamics to the quantitative data of biochemical cycles in cells.

## Footnotes

a τ_f_^crowded^ = τ_f_^aq^η_eff_/η_0_.

b To calculate this ratio, we first determined the hydrodynamic drag of an unloaded F_1_ domain rotating diffusively along its long axis. We assumed the domain to be a cylinder with a radius *r* of 5.23 nm and a height *h* of 15.6 nm based on molecular distances of the residues from Protein Data Bank entry 6N2Z.^21^ The hydrodynamic drag for such an object is given as ζ = 4*πη*_0_*hr*^2^. The hydrodynamic drag of the loaded motor was calculated according to the procedure described in ref (18).

c Also known as the Brownian motor.^23^

d For the purpose of this work, we will use “*ex vivo*” for material extracted from living cells and “*in vivo*” for measurements performed inside living cell (including cells in culture).
